# Growing and equipping the infection control community in Singapore

**DOI:** 10.1186/1753-6561-5-S6-P4

**Published:** 2011-06-29

**Authors:** ML Ling, H Oh, SL Lee, SC Wong, L Lang, Infection Control Association of Singapore

**Affiliations:** 1 Infection Control, Singapore General Hospital, Singapore, Singapore; 2 Medicine, Changi General Hospital, Singapore, Singapore; 3 Infection Control, Thomson Medical Centre, Singapore, Singapore; 4 Infection Control, Khoo Teck Puat Hospital, Singapore, Singapore; 5 School of Nursing, Parkway College, Singapore, Singapore

## Introduction / objectives

Infection Control Association (Singapore), ICAS, founded in 1999, has 153 members of whom 25 are from countries outside Singapore. To facilitate exchange of information and data on infection control principles and practices, ad-hoc seminars and workshops are held to address topics of interest.

## Methods

It conducted its 1^st^ basic course in Infection Control in 2002, in collaboration with the Asia Pacific Society of Infection Control (APSIC). This comprehensive program attracted large number of ICPs from the region and is offered once in 2 years. An Advance Course is held on alternate years to meet needs of senior ICPs. ICAS developed 3 national guidelines or standards through consensus working groups from its members and the Ministry of Health – Schools and Day Care Centres; Intermediate and Long Term Care Centres and the Management of MRSA Patients.

## Results

In 2009, it celebrated its 10^th^ Anniversary with the launch of its 1^st^ International Congress of ICAS, which helped to foster research in the Infection Control Community. Its recently held 2^nd^ congress was a success with close to 400 registrants for its congress and post-congress workshops.

## Conclusion

The effectiveness of infection control programs now demand that our ICPs turn to creative strategies to achieve improved clinical outcomes with conserved resources and lower cost. The challenge for ICAS in the next decade will be to equip its members to be effective agents of change.

## Disclosure of interest

None declared.

